# Heated Ultrasound Gel and Patient Satisfaction with Bedside Ultrasound Studies: The HUGS Trial

**DOI:** 10.5811/westjem.2017.8.35606

**Published:** 2017-09-11

**Authors:** Benjamin M. Krainin, Lane C. Thaut, Michael D. April, Ryan A. Curtis, Andrea L. Kaelin, Garrett B. Hardy, Wells L. Weymouth, Jonathan Srichandra, Eric J. Chin, Shane M. Summers

**Affiliations:** San Antonio Uniformed Services Health Education Consortium, Department of Emergency Medicine, San Antonio, Texas

## Abstract

**Introduction:**

Our goal was to determine if heated gel for emergency department (ED) bedside ultrasonography improves patient satisfaction compared to room-temperature gel.

**Methods:**

We randomized a convenience sample of ED patients determined by their treating physician to require a bedside ultrasound (US) study to either heated gel (102.0° F) or room-temperature gel (82.3° F). Investigators performed all US examinations. We informed all subjects that the study entailed investigation into various measures to improve patient satisfaction with ED US examinations but did not inform them of our specific focus on gel temperature. Investigators wore heat-resistant gloves while performing the examinations to blind themselves to the gel temperature. After completion of the US, subjects completed a survey including the primary outcome measure of patient satisfaction as measured on a 100-mm visual analogue scale (VAS). A secondary outcome was patient perceptions of sonographer professionalism measured by an ordinal scale (1–5).

**Results:**

We enrolled 124 subjects; 120 completed all outcome measures. Of these, 59 underwent randomization to US studies with room-temperature gel and 61 underwent randomization to heated US gel. Patient 100-mm VAS satisfaction scores were 83.9 among patients undergoing studies with room-temperature gel versus 87.6 among subjects undergoing studies with heated gel (effect size 3.7, 95% confidence interval −1.3–8.6). There were similarly no differences between the two arms with regard to patient perceptions of sonographer professionalism.

**Conclusion:**

The use of heated ultrasound gel appears to have no material impact on the satisfaction of ED patients undergoing bedside ultrasound studies.

## INTRODUCTION

### Background

Patient satisfaction is an increasing outcome of interest for emergency department (ED) providers.^1^–^4^ Hospital administrators increasingly scrutinize satisfaction scores and link results with physician reimbursement.^5^ Moreover, there exists a correlation between ED visit satisfaction scores and the likelihood of patients filing complaints related to their care. In one study of over 2.4 million ED visits across eight different states, patients who responded in the lowest quartile of satisfaction scores were twice as likely to file a complaint compared to the patients with satisfaction scores in the uppermost quartile.^6^

Bedside ultrasound (US) is a diagnostic tool rapidly increasing in use by ED providers.^7^,^8^ In the hands of emergency physicians at the bedside, this modality has shown high sensitivity and specificity for the diagnosis of myriad common diseases encountered in the ED such as appendicitis,^9^ cholecystitis,^10^,^11^ and deep vein thrombosis.^12^ There is further an association between the use of bedside ultrasonography and increased patient satisfaction scores.^13^ However, to date there has been little research to elucidate those components of bedside ultrasonography with the strongest relationship to patient satisfaction.

US gel temperature represents one important component of bedside ultrasonography. Many US technicians routinely use heated US gel to enhance patient comfort. Several gel warmer class I medical devices exist, which may provide an effective and inexpensive mechanism to heat US gel for this purpose. However, our anecdotal experience is that many EDs do not routinely use these devices. To our knowledge, no studies exist that examine the impact of heated gel on satisfaction scores among patients undergoing US studies.

### Study Objectives

The primary objective of this investigation was to determine if heated gel for ED bedside ultrasonography improves patient satisfaction compared to room-temperature gel. The secondary objective was to determine the impact of heated US gel use on patient perceptions of ultrasonographer professionalism. We hypothesized that the use of heated gel during bedside US examinations would improve patient satisfaction scores and perceptions of provider professionalism.

## METHODS

### Study Design and Setting

We conducted a randomized controlled trial in the ED of an academic, urban, tertiary care hospital. The ED annual census is approximately 82,000 visits. The ED supports a three-year emergency medicine residency and fellowship programs in US and emergency medical services. Our institutional review board (IRB) approved the project. We registered the trial on ClinicalTrials.gov (NCT03135379), and documented subject participation in accordance with the CONSORT guidelines (Figure).^14^

### Selection of Participants

We enrolled a convenience sample of adult ED patients. Inclusion criteria comprised patients determined by their treating provider to require a bedside US study delineated by the American College of Physicians as falling within the scope of practice for emergency physicians.^15^ Exclusion criteria included age less than 18, age greater than 89, pregnancy, skin lesions precluding bedside US examination, or patients not fluent in English. We also excluded vulnerable patient populations, specifically patients with altered mental status, prisoners, and military basic trainees.

**Population Health Research Capsule**

What do we already know about this issue?

Bedside ultrasound is rapidly increasing in use by emergency department (ED) providers. Methods of performing these exams may impact patient satisfaction.

What was the research question?

How does heated versus room-temperature gel for ED bedside ultrasonography affect patients’ satisfaction?

What was the major finding of the study?

ED ultrasonography gel temperature does not significantly impact patients’ satisfaction with their ED visits.

How does this improve population health?

This negative result suggests that providers seeking to improve ED patient satisfaction should focus on alternative targets aside from ultrasound gel temperature.

All subjects received an information sheet disclosing that they would participate in a study investigating alternative strategies to improve patient satisfaction related to US studies. While we could not blind subjects to gel temperature, we did not disclose to patients that the primary purpose of the study was to investigate the impact of US gel temperature on patient satisfaction. All subjects provided verbal consent for study participation. Our IRB approved this alteration of the consent process and waiver of documentation of informed consent as they determined the research was minimal risk to participants, did not adversely affect the rights of subjects, would not be practical without these provisions, and allowed for provision of pertinent information to participants when appropriate.^16^

### Interventions

We randomized patients to either warm gel (102.0° F) or room-temperature gel for the bedside ultrasonography examinations. Prior to study start, investigators constructed a workstation in the ED with six standard gel-warming devices (Thermasonic® Gel Warmer, Parker Laboratories, Fairfield, NJ). We configured three of the six devices to heat the gel to 102.0° F. We turned the remaining three devices off, allowing the gel to remain at room temperature. We validated gel temperature through weekly quality assurance measurements throughout the study period. The mean of these temperature measurements for the heated ultrasound gel was 102.0° F, whereas that for the room temperature gel was 82.3° F. We obtained these measurements by a Suretemp® Plus 692 Thermometer (Welch Allyn Inc., Skaneateles Falls, NY). The ambient temperature maintained in our ED is 70.0° F. We assigned each of the six devices a unique identification number. The study packet for each participant included a card with the identification number of the gel warmer to which we allocated that particular subject. We used a simple randomization sequence to allocate subjects to each study arm.

Eight emergency medicine resident and US fellow investigators performed all ultrasonography examinations. They performed all US examinations using one of four identical Sonosite M-Turbo® ultrasound machines (Fujifilm Sonosite, Inc., Bothell, WA). Investigators would retrieve any of these four devices based on device availability for use during study procedures: there was no systematic allocation of devices according to study arm. We stored the US machines separately from the gel-warming devices.

We took several measures to blind investigators to the temperature of the gel used for each subject. First, we obscured the power indicator light on the gel-warming devices with non-transparent tape. In addition, the investigators wore a heat-resistant glove (ULine terry cloth glove, Pleasant Prairie, WI) during the entirety of the study procedures, starting with retrieval of the gel from the assigned warmer. During the US examinations, the investigators additionally wore a sterile non-latex glove (synthetic polyisoprene surgical gloves, Molnlycke Health Care Pty Ltd, Norcross, GA) over the heat-resistant glove for infection control purposes; at no time did the investigators remove the heat-resistant glove during the study procedures. These methods aimed to maintain blinding even in the event that reapplication of gel during the US examination was necessary. The heat-resistant glove resists temperatures up to 250° Fahrenheit. We validated the efficacy of this glove for maintaining blinding to gel temperature prior to study start. Specifically, 10 volunteers not otherwise affiliated with the study donned the gloves while simulating bedside US procedures on a manikin model. We provided five of these volunteers warmed gel and five with room-temperature gel. None of the volunteers successfully identified the temperature of the gel.

### Outcome Measurements

We measured study outcomes with hard-copy surveys upon conclusion of study procedures, which solicited patient demographics (age and gender). The survey administered to patients comprised three questions. The first was, “How satisfied are you with the experience of having a bedside ultrasound today?” The response to this first question comprised a 100-mm visual analogue scale (VAS) for satisfaction as used in previous studies.^17^ This response comprised the primary outcome for the study. Responses to the remaining questions on the subject survey comprised secondary outcomes. These questions included, “Are you satisfied with the care you received today in the emergency department?” The response to this question was binary (yes or no). The final subject survey question read, “How professional was the provider who performed your bedside ultrasound?” The response to this final question comprised a Likert scale spanning 1 (“very unprofessional”) to 5 (“very professional”).

Upon completion of study procedures for each subject, we also administered a hard-copy survey to each investigator performing the study US examination. The purpose of this survey was to ascertain the effectiveness of our blinding methodology. Specifically, the survey question read, “What was the temperature of the ultrasound gel you used during the study?” Response options were “warm,” “room temperature,” or “I do not know.”

### Analysis

Our sample size estimate used an alpha of 0.05 and a beta of 0.2. Based on previous ED-based studies using a patient satisfaction VAS we powered our study to detect a minimally clinically significant difference of 11 mm.^18^ We anticipated a standard deviation of 21 mm based on internal quality-improvement data. Our estimated required sample size to detect this effect size was 114 participants. With an additional estimated 10 subject withdrawals or dropouts, our total enrollment requirement was 124 participants.

We double-entered all hard-copy data forms into a secured Excel database (version 14, Microsoft, Redmond, WA). We then exported all data into SPSS (version 21, IBM, Armonk, NY) for statistical analysis. We excluded subjects with missing data for the primary outcome from all analyses. We compared our primary outcome of satisfaction VAS using a two-tailed independent samples student t-test. And we compared our secondary outcome of perceived professionalism with a Mann-Whitney U test. We planned comparison of our secondary outcome of overall satisfaction with ED care (binary variable) with a chi-squared test. Finally, we compared provider responses to the inquiry regarding whether the gel for each patient was room temperature, warmed, or uncertain using a chi-squared test.

## RESULTS

### Study Subject Characteristics

All 124 patients screened for enrollment were eligible for participation and verbally consented to the study. Half (62) of these subjects underwent allocation to room-temperature gel while the remaining subjects underwent allocation to heated gel. No patients withdrew from the study prior to completion. Survey data were incomplete for three subjects in the room-temperature arm and one subject in the heated-gel arm. This resulted in 59 subjects for analysis in the room-temperature arm and 61 subjects for analysis in the heated-gel arm (Figure).

Patient baseline characteristics including age and sex were comparable between the two groups (Table 1). Studies performed were diverse and included focused assessment with sonography in trauma and US studies of the kidneys, aorta, gallbladder, gastrointestinal tract (e.g., appendix, hernias), heart, eyes, skin, bones, and testicles.

### Main Results

Mean patient 100-mm VAS satisfaction scores were 83.9 (standard deviation 15.5) among patients undergoing studies with room-temperature gel vs. 87.6 (standard deviation 10.5) among subjects undergoing studies with heated gel (effect size 3.7, 95% confidence interval −1.3–8.6, Table 2). All subjects in both arms reported satisfaction with regard to their ED visit. There were similarly no differences between the two groups with regard to the secondary outcome of perceived investigator professionalism.

Provider responses to the inquiry regarding whether the gel for each patient was room temperature, warmed, or uncertain indicated imperfect investigator blinding (Table 3). Investigators reported the correct gel temperature for 21 of 59 (36%) subjects undergoing US studies with room temperature gel. Investigators reported the correct gel temperature for 16 of 61 (26%) subjects undergoing US studies with warmed gel.

## DISCUSSION

Use of bedside US imaging in the ED is on the rise.^7^ Simultaneously, interest continues to grow in the emergency medicine literature with regard to investigations of interventions to improve patient satisfaction.^1^–^3^ To our knowledge, ours is the first and only study to examine the impact of heated gel on patient satisfaction. Our results indicate that heated US gel has no material impact on the satisfaction of ED patients undergoing bedside US studies.

Our study provides high-quality evidence in support of this conclusion. Our randomized design should eliminate the impact of confounders on our results. While we did observe a trend toward slightly higher patient satisfaction with heated gel, the effect size did not reach the minimally clinically significant differences for patient satisfaction reported in the literature (7–11 mm).^18^ Similarly, we observed no significant differences based on US gel temperature with regard to patient overall satisfaction with their ED visit, or patient perceptions of emergency physician professionalism.

These findings will likely be welcome to many ED administrators, given our anecdotal experience that few EDs use gel warmers. While gel warmer devices are relatively inexpensive, we surmise their limited use in ED settings largely relates to the dynamic nature of ED care. Whereas gel warmer devices generally require a power outlet and must generally remain in a static location, US machines in the ED are portable and frequently moved to various rooms throughout the department. Given the time demands placed upon the emergency physician, it is often impractical to return to a fixed location between examinations to retrieve warmed gel.

It is important to highlight that these results may not be generalizable outside of the ED setting. We expect the ED population to present with more acute illness, discomfort, and anxiety than patients in office-based practices. While our inclusion criteria required that patients not be so critically ill as to compromise their mental status to provide verbal consent, it is possible that their levels of pain and anxiety precluded them from focusing on the discomfort associated with the US gel temperature. Future studies repeating our study methodology in alternative patient populations may yield different results. It is also possible that future studies conducted with different internal climates resulting in different room temperature values would yield different results.

## LIMITATIONS

Our study has several important limitations. First, the post-study procedures investigator survey responses suggested imperfect investigator blinding to the gel temperature. This occurred despite our efforts to maintain blinding by having investigators wear heat-resistant gloves during the entirety of the study procedures for each patient. The most likely reason for this finding is patient verbal or physical response following application of gel. Anecdotally, numerous patients flinched upon application of room-temperature gel to their skin. Conversely, other patients commented upon the pleasant sensation of the heated gel. Another possibility is that the decrease in gel viscosity following heating may potentially have allowed investigators to ascertain the temperature of the gel being used. However, given that all investigators were privy to the study hypothesis, this lack of blinding threatens a Type I error (false-positive study result), which we know did not occur given our negative study result.

Second, our study population is the product of convenience sampling. Consequently, our study may suffer from sampling bias. For example, investigators may have preferentially enrolled patients who appeared less uncomfortable and more amenable to study participation. In so doing, we may have enrolled a study population likely to express satisfaction with study and ED procedures regardless of intervention, blunting our measured effect size for our primary outcome. Similarly, investigators may have preferentially enrolled patients with body compositions more amenable to ultrasonography. This form of sampling bias may have led to less overall discomfort associated with the US examinations among a relatively homogenous patient population.

A related limitation is the timing of survey completion. As investigators requested that subjects complete the survey immediately upon completion of the study procedures, patients may have felt compelled to offer more favorable survey responses given the temporal proximity to the US examination and physical presence of the investigator in the room. This approach to data collection could blunt any effect size measurements by leading to uniformly high satisfaction responses from subjects regardless of the intervention to which they were allocated. While more laborious and logistically challenging, future studies might avoid this potential problem by administering the surveys at the end of the ED stay without any study personnel present instead of right after completion of study procedures.

A fourth limitation is that we did not mandate a particular type of training scan for investigators to complete (e.g. gallbladder, ocular, etc.) Study type may be an important effect modifier for the impact of gel temperature on patient satisfaction given that some studies require more time or involve more sensitive areas of the body than others. A related limitation is our exclusion of pregnant women, many of whom undergo transvaginal US examinations in the ED for which gel temperature may have a greater impact on patient satisfaction. We also did not collect comprehensive data regarding patient characteristics (e.g., age, gender) or operator experience (e.g., years of post-graduate training, numbers of prior US studies). Future studies might consider repeating our study procedures with either larger patient numbers for stratified analyses or narrower inclusion/exclusion criteria and homogenous operators to ascertain whether gel temperature might be a more potent driver of patient satisfaction under various specific circumstances.

A fifth limitation is that our study only examined a single set of temperatures. Specifically, our quality assurance measurements found mean temperatures of 102.0° F for the warmed gel and 82.3° F for the room-temperature gel. It is possible that changes in these temperature values could result in different results than those found in our study. To the extent that there is a greater differential between the temperatures of warmed and non-warmed gel, we anticipate that gel temperature may have a greater impact on patient satisfaction.

Another important point is that our study outcome measure examines overall visit satisfaction. Consequently, we cannot speak to whether US gel temperature impacts satisfaction specifically with regards to ED US examinations. Our reasoning for instead focusing on overall visit satisfaction is that this represents a more definitive and terminal outcome. One might argue that should heated US gel increase patient satisfaction with ED US studies but not overall ED visit satisfaction that there is limited justification for implementing this intervention. Nevertheless, future studies might consider examining the impact of US gel temperature on the more proximal and immediate outcome of satisfaction with US examination.

A final limitation is a lack of reporting on several additional outcome measures of potential interest to emergency physicians. In particular, such outcome measures would include sonographer time at bedside and patient ED length of stay. Future investigations in this area should consider incorporation of these additional outcomes.

All these limitations notwithstanding, our results suggest that investment in ED gel-warming devices should be low priority. On the other hand, given that the expense is likely to have a minimal impact on overall operating budget, if future studies were to identify a beneficial effect of heated gel on patient satisfaction ED administrators should have a low threshold for obtaining these machines as they would likely have relatively low cost-to-benefit ratios.^19^

## CONCLUSION

Although there is a trend towards increased patient satisfaction with heated ultrasound gel, the effect size appears to be insignificant. Researchers might consider focusing future investigations on more specific settings and ultrasound study types for which gel temperature is more likely to impact patient comfort and hence satisfaction. In the interim, emergency physicians looking to improve patient satisfaction are likely to have more success focusing on more traditional targets, such as decreasing patient wait times rather than using gel-warming devices.

## Figures and Tables

**Figure:**
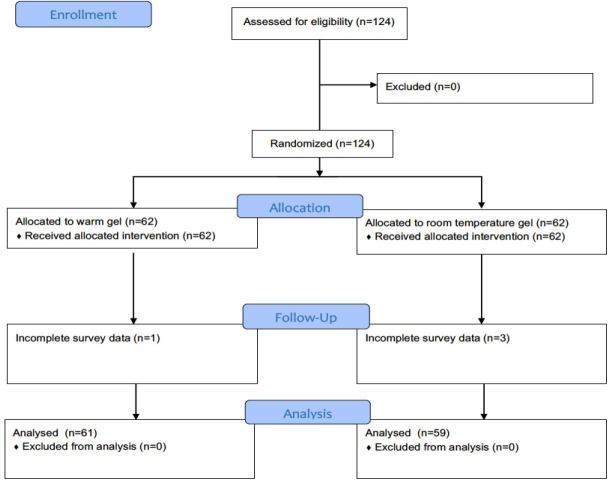
Consort 2010 Flow Chart. Patient trial participation in study examining patient satisfaction as related to temperature of ultrasound gel.

**Table 1:** Patient characteristics.

Variable	Room temperature (n=59)	Warmed (n=61)
Mean age, years (95% CI)	42.0 (37.2–47.1)	41.5 (37.2–45.9)
Male sex, % (95% CI)	46 (33–58)	69 (56–81)

*CI*, confidence interval.

**Table 2:** Outcomes of study examining impact of heated vs. room-temperature ultrasound gel on patient satisfaction.

Variable	Room temperature (n=59)	Warmed (n=61)	Effect size
Mean VAS satisfaction score (95% CI)	83.9 (79.4–87.6)	87.6 (84.8–90.1)	3.7 (−1.3–8.6)
Median professionalism score (IQR)	5 (5–5)	5 (5–5)	0 (0–0)

*CI*, confidence interval; *IQR*, interquartile range; *VAS*, visual analogue scale.

**Table 3:** Blinding efficacy.

Investigator-reported gel temperature	Actual gel temperature	

	Room temperature (n=59)*	Warmed (n=61)
Room temperature	21	1
Warmed	0	16
Unsure	37	44

* Data missing for one subject.

## References

[1] Beadle KL, Helbling AR, Love SL (2016). Isopropyl alcohol nasal inhalation for nausea in the emergency department: a randomized controlled trial. Ann Emerg Med.

[2] Bendesky BS, Hunter K, Kirchhoff MA (2016). Same physician, different location, different patient satisfaction scores. Ann Emerg Med.

[3] Handel DA, French LK, Nichol J (2014). Associations between patient and emergency department operational characteristics and patient satisfaction scores in an adult population. Ann Emerg Med.

[4] McCarthy ML, Ding R, Zeger SL (2011). A randomized controlled trial of the effect of service delivery information on patient satisfaction in an emergency department fast track. Acad Emerg Med.

[5] Geiger NF (2012). On tying Medicare reimbursement to patient satisfaction surveys. Am J Nurs.

[6] Cydulka RK, Tamayo-Sarver J, Gage A (2011). Association of patient satisfaction with complaints and risk management among emergency physicians. J Emerg Med.

[7] Levin DC, Rao VM, Parker L (2014). Continued growth in emergency department imaging is bucking the overall trends. J Am Coll Radiol.

[8] Smith-Bindman R, Aubin C, Bailitz J (2014). Ultrasonography versus computed tomography for suspected nephrolithiasis. N Engl J Med.

[9] Gungor F, Kilic T, Akyol KC (2017). Diagnostic value and effect of bedside ultrasound in acute appendicitis in the emergency department. Acad Emerg Med.

[10] Summers SM, Scruggs W, Menchine MD (2010). A prospective evalua tion of emergency department bedside ultrasonography for the detection of acute cholecystitis. Ann Emerg Med.

[11] Villar J, Summers SM, Menchine MD (2015). The absence of gallstones on point-of-care ultrasound rules out acute cholecystitis. J Emerg Med.

[12] Pomero F, Dentali F, Borretta V (2013). Accuracy of emergency physician-performed ultrasonography in the diagnosis of deep-vein thrombosis: a systematic review and meta-analysis. Thromb Haemost.

[13] Howard ZD, Noble VE, Marill KA (2014). Bedside ultrasound maximizes patient satisfaction. J Emerg Med.

[14] Schulz KF, Altman DG, Moher D (2010). CONSORT 2010 statement: updated guidelines for reporting parallel group randomized trials. Ann Intern Med.

[15] Bellamkonda VR, Shokoohi H, Alsaawi A (2015). Ultrasound credentialing in North American emergency department systems with ultrasound fellowships: a cross-sectional survey. Emerg Med J.

[16] Ernst AA, Fish S (2005). Exception from informed consent: viewpoint of institutional review boards--balancing risks to subjects, community consultation, and future directions. Acad Emerg Med.

[17] Counselman FL, Graffeo CA, Hill JT (2000). Patient satisfaction with physician assistants (PAs) in an ED fast track. Am J Emerg Med.

[18] Singer AJ, Thode HC (1998). Determination of the minimal clinically significant difference on a patient visual analog satisfaction scale. Acad Emerg Med.

[19] April MD, Murray BP (2017). Cost-effectiveness analysis appraisal and application: an emergency medicine perspective. Acad Emerg Med.

